# Diagnosis of Klippel-Trenaunay syndrome and extensive heterotopic ossification in a patient with a femoral fracture: a case report and literature review

**DOI:** 10.1186/s12891-020-03224-2

**Published:** 2020-04-11

**Authors:** Wanbo Zhu, Kai Xie, Jiazhao Yang, Li Li, Xujin Wang, Lei Xu, Shiyuan Fang

**Affiliations:** 1 Affiliated Anhui Provincial Hospital of Anhui Medical University, Hefei, 230001 Anhui China; 2 Department of Orthopedics, The First Affiliated Hospital of USTC, Division of Life Sciences and Medicine, University of Science and Technology of China, Hefei, 230001 Anhui China

**Keywords:** Klippel-Trenaunay syndrome, Heterotopic ossification, FoxO1

## Abstract

**Background:**

Klippel-Trenaunay syndrome (KTS) is a rare complex vessel malformation syndrome characterized by venous varicosities, capillary malformations, and limb hypertrophy. However, extensive heterotopic ossification (HO) secondary to this syndrome is extremely rare.

**Case presentation:**

We report the case of a patient with previously undiagnosed KTS and extensive HO who presented with a femoral fracture secondary to a motor vehicle accident. Extensive ossification, which leads to compulsive contracture deformity and dysfunction of the leg, was distributed on the flexor muscle side, as revealed by the radiograph. The diagnosis was finally established by combining imaging and histological analysis with classical clinical symptoms. Amputation was performed at the fracture site proximal to the infected necrotic foci. Open management of the fracture was challenging owning to the pervasive ossification and tendency for excessive bleeding. Gene sequencing analysis showed homozygous mutation of FoxO1 gene.

**Conclusions:**

Definitive diagnosis of a combination of KTS and extensive HO requires detailed imaging analysis and pathologic evidence. Mutation of the FoxO1 gene, which regulates bone formation by resistance to oxidative stress in osteoblasts, is a potential factor in the microenvironment of malformed vessels caused by KTS.

## Background

Klippel-Trenaunay Syndrome (KTS) is a rare congenital disorder of the vascular system that is usually diagnosed at birth. It mostly occurs in one limb in the lower extremities and is characterized by a clinical triad of a) capillary malformations (port wine stain); b) varicose veins or venous malformations; and c) soft tissue and bone hypertrophy [1]. Clinical diagnosis requires the presence of at least two of these signs. When arteriovenous malformations coexist, Klippel-Trenaunay-Weber syndrome (or Parkes-Weber) is diagnosed [2]. Fracture of the long bones in KTS requires careful management owing to profuse bleeding, non-healing wounds, infection, and non-union of the bone [3, 4].

Heterotopic ossification (HO) is pathological bone formation at extra-skeletal sites, such as soft tissues, which limits bone and joint activity [5]. HO is often divided into the acquired non-genetic form and inherited genetic form. Extensive HO is often caused by severe trauma or due to inherited conditions, such as fibrodysplasia ossificans progressiva or progressive osseous heteroplasia [6]. However, to the best of our knowledge, there are no published reports of HO combined with KTS or HO secondary to KTS.

We describe a rare case of subtrochanteric femoral fracture in a patient with KTS and extensive HO in the leg.

## Case presentation

**Fig. 1:**
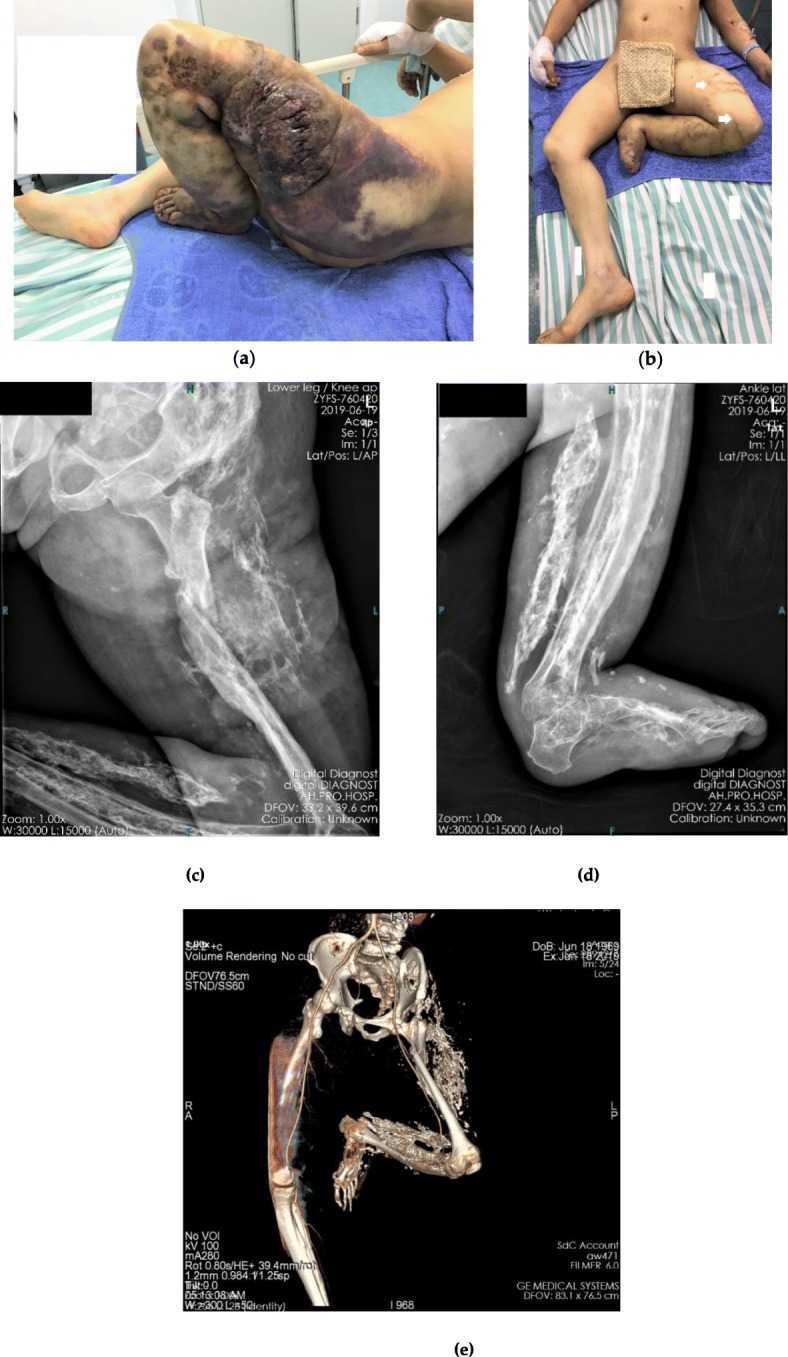
Photographs and images of the injury. **a** Initial presentation of hemangioma, a 15-cm stitched wound can be seen on the hemangioma; **b** Contracture of the leg, the white arrows show superficial varicose veins; **c**-**d** Radiograph showing femoral fracture and extensive high-density shadows distributed at the flexor muscle side of left lower extremity; **e** Three-dimensional computed tomography (3DCT). Vascular reconstruction shows coherence and integrity of the main arteries of the lower extremities

A 46-year-old man was transferred to our emergency department with a 15-cm stitched wound on a large kermesinus hemangioma-like lump on the left lateral thigh (Fig. 1a). The patient was hit by a car 1 day before. The patient was admitted immediately. The entire left lower extremity presented a compulsive contracture deformity with massive swelling, induration, and varicosities (Fig. 1b). Systemic examination revealed severe anemia, and an emergency transfusion was performed. Radiographs of the left leg showed a shaft fracture at the proximal third of the femur with extensive high-density shadows distributed in the flexor muscle side (Fig. 1c-d). Ultrasonography of the limb showed normal blood flow of the main vessels and venous malformations in the dermis. Three-dimensional computed tomography (CT) reconstruction confirmed a femoral fracture and a continuous artery with massive skeletal structural deformities along the extremity (Fig. 1e). The contralateral limb radiograph revealed a normal skeletal structure.

**Fig. 2:**
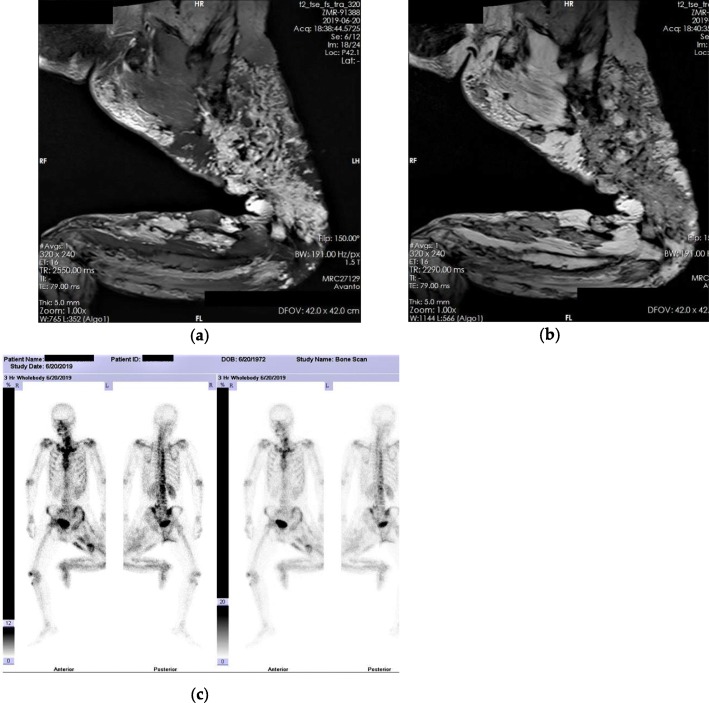
Additional imaging studies. **a**-**b** Magnetic Resonance Imaging (MRI) shows extensive high signal foci within the muscles; **c** Technetium 99 m-methyl diphosphonate (99mTc-MDP) bone scan shows radioactivity concentration at the flexor side of the limb

The patient claimed to have a “port-wine birthmark” on the left foot at birth. With growth and development, the port-wine stain started to spread, and the entire left lower limb was progressively flexed and contracted with loss of function. The condition stabilized after his growth stopped. Magnetic resonance imaging (MRI) suggested diffuse soft tissue hemangiomas, and a diagnosis of KTS was made based on the medical history and clinical characteristics (Fig. 2a and b). A technetium 99 m-methyl diphosphonate (99mTc-MDP) bone scan also indicated extensive radioactivity concentration on the flexor side of the limb, and a diagnosis of HO was inferred (Fig. 2c). However, it was difficult to distinguish whether the high-density shadow and radioactive concentration exhibited on imaging was intramuscular calcification or ossification.

**Fig. 3:**
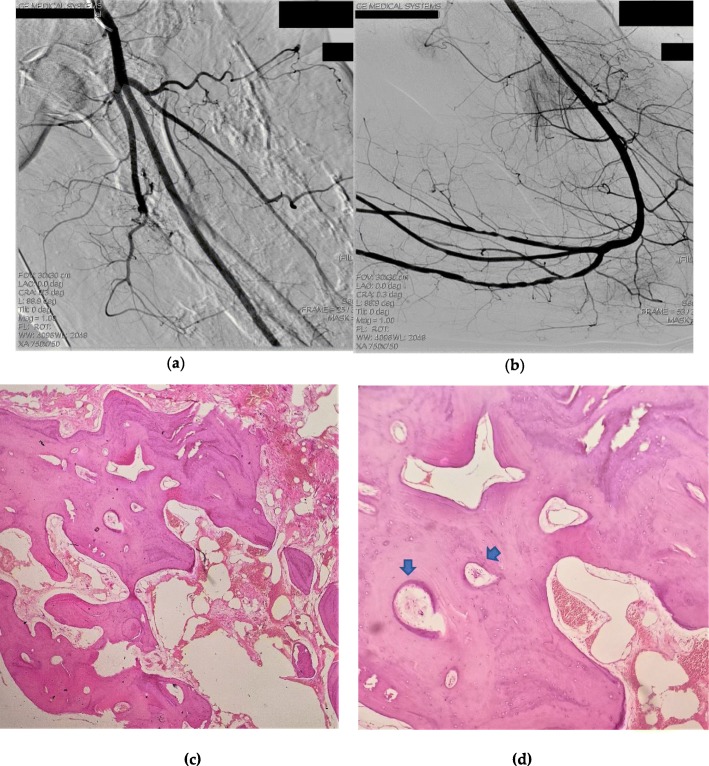
Necrosis and surgical outcome. **a**-**b** Lower extremity digital subtraction angiography shows vascular distribution. **c**-**d** Pathological specimens of ossification shows trabecular bone formation and bone structure construction. **c** magnification × 100 and **d** magnification × 40 (Hematoxylin & Eosin staining). The blue arrows show osteocytes

**Fig. 4:**
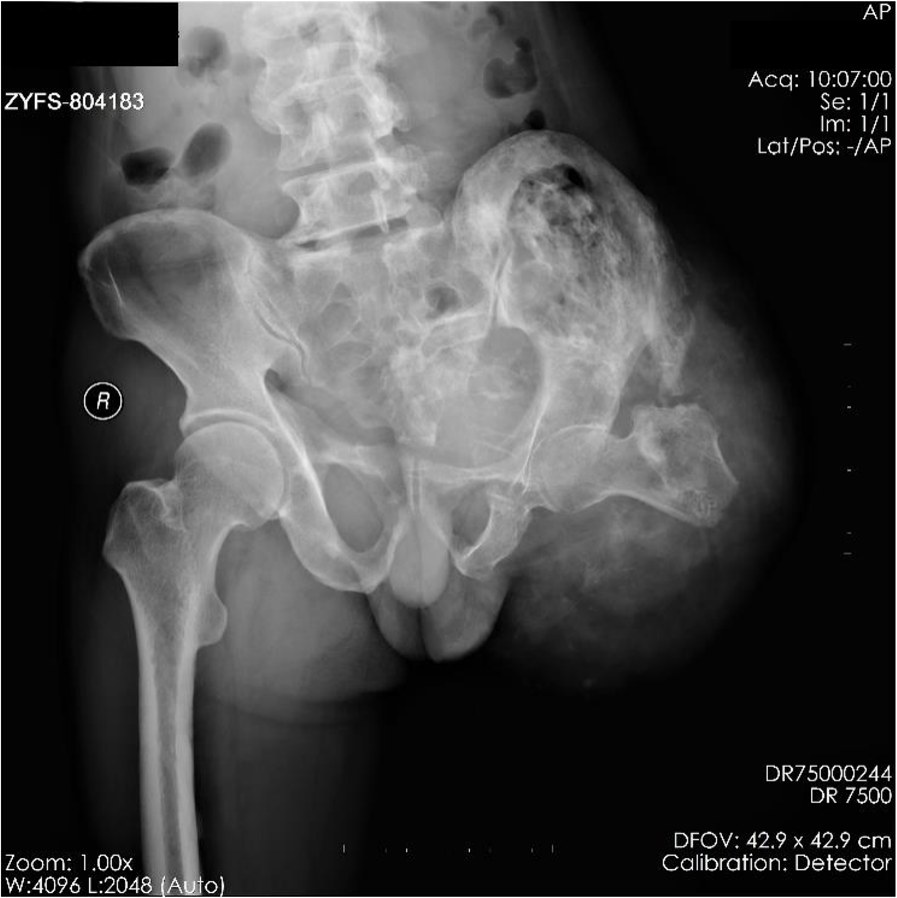
Postoperative X-ray showed fracture site after amputation

After hospitalization, the patient’s lateral thigh wound began to show signs of necrosis on the 3rd day. Lower extremity digital subtraction angiography (DSA) showed vascular distribution and blood supply of the diseased limb (Fig. 3a-b). No arteriovenous fistulas were found during DSA. On the fifth day, bacterial culture from the wound showed a gram-positive bacterial infection. On the seventh day, an amputation was performed at the fracture site to the proximal of the infected necrotic foci. Owing to the high amputation plane, a tourniquet could not be used. Due to extensive vascular malformations and soft tissue ossification, bleeding during surgery was excessive and difficult to control by conventional electrocoagulation and ligation. Extensive ossification impeded the progress of the surgery. The intraoperative blood transfusion was 17 units. The patient was transferred to the intensive care unit (ICU) for advanced life support. Ossification specimens provided histopathologic evidence of HO (Fig. 3c-d). Normal trabecular bone formation and bone structure construction were detected. The patient was discharged on the 33rd day after hospitalization, and a postoperative X-ray was performed (Fig. 4). In order to maintain hemoglobin stability, the patient was transfused 55 units of blood during hospitalization. On follow-up at 2 months, the amputation wound healed well.

**Fig. 5:**
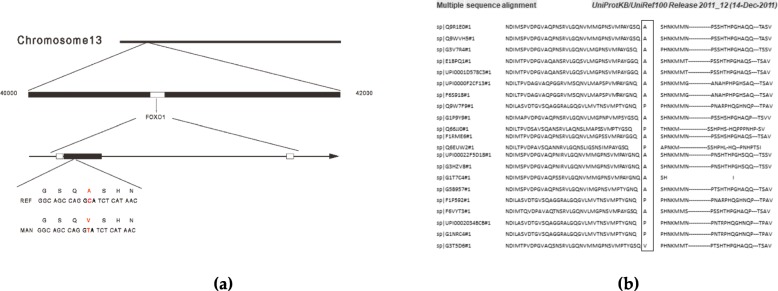
Gene sequencing analysis of Foxo1. **a** The idiogram of Foxo1 translation error at chromosome 13. **b** Sequence alignment shows the A551 is identical among different species

The patient and his family provided informed consent for genetic testing. Gene sequencing was performed using next-generation sequencing technology (NGS). Heterozygous mutations at chromosome 13 in both parents led to a homozygotic mutation in the patient, resulting in a FoxO1 translation error (Fig. 5a-b). The sequencing results were explained to the subjects according to the American College of Medical Genetics and Genomics guidelines [7]. The study was approved by the local ethics committee.

The NGS sequencing of the FoxO1 gene revealed a novel homozygotic missense variation in Exon 2, c.1532C > T, leading to a translation error at amino acid 551 (p.A551V). It was predicted to be disease causing based on Sorting Intolerant From Tolerant (SIFT) analysis. The SIFT score was 0.041, and the SIFT converted rank score was 0.419.

## Discussion and conclusions

In this study, we report the case of a patient with a femoral fracture secondary to a motor vehicle accident diagnosed with KTS following imaging and histological analysis. Mutation of the FoxO1 gene, which regulates bone formation by resistance to oxidative stress in osteoblasts, is a potential factor in the microenvironment of malformed vessels caused by KTS.

Vascular malformations in KTS usually affect the capillary, venous, and lymphatic systems of the lower extremities, which leads to swelling, varices, and ulcerations of the diseased limb [1]. Elevated D-dimer levels and mutation of the AGGF1 gene are considered to suggest the diagnosis [8]. However, owing to coagulation disorders caused by the fracture in our case, D-dimer was high during hospitalization and could not be considered suggestive of KTS [9]. Whole genome sequencing did not reveal AGGF1 or PIK3CA mutations. MRI is essential for the diagnostic evaluation of KTS as it reveals differences in the vascular malformations and soft tissues [10]. Multiple high signal foci within the muscles can be seen on T2 SE sequences. In addition, duplex ultrasound imaging and DSA provide indirect and direct evidence of vascular morphology and function, providing references for diagnosis.

Clinical diagnosis of KTS depends on the classic presentation [11]. Clinical presentation of KTS in our case was typical; however, flexion deformity of the lower extremities is not common. We found a case of KTS with lower extremity contracture, but no ossification was found on MRI and CT [10]. The author attributed the contracture to muscle atrophy and disuse. In this case, the presence of extensive ossification of the lower extremity flexor may have caused lower extremity contracture. Diagnosis of HO mostly depends on radiographic imaging and clinical history. CT, single photon emission CT (SPECT), and bone scanning may help to identify the extent of ossification and aid in early detection [12]. However, the gold standard for HO diagnosis is still the pathological results of tissue biopsy suggesting bone trabecular growth and bone structure formation [13].

No inherited HO-related gene mutation was found using whole exome sequencing. No history of traumatic head or spinal cord injury was claimed. No possible pathogenic lesion was detected on brain or spinal MRI. The combination of clinical symptoms and history, suggested the diagnosis of acquired HO. Acquired HO refers to abnormal bone tissue outside the normal skeletal system. Uncontrolled signal transduction plays a key role in recruiting and inducing the differentiation of progenitor cells such as mesenchymal stem cell and mesenchymal progenitor cell, promoting bone formation and remodeling [5]. Acquired HO is mostly induced by orthopedic trauma or neurogenic injuries [14]. Hypoxia and inflammation are associated with the episodic induction of HO [15]. Hypoxia-inducible factor-1α inhibits fusion of inclusion body regulated by rabaptin5, thereby, regulating intracellular BMP receptor activity and activating BMP signaling pathway [16]. The formation of HO can be induced through both BMP/Smad pathway and BMP/P38 MAPK pathway [17–19]. In addition, signaling pathway including Hedgehog, Wnt-β-catenin and NF-κB also contribute to formation of HO [20–22]. In our case, a local hypoxic environment caused by the extensive hemangioma and vascular malformation is a possible mechanism for inducing ossification [23]. The inflammatory response caused by small trauma in the capillary network is also a potential predisposing factor. Although KTS belongs to the PIK3CA-related overgrowth spectrum, we did not find mutations in the PIK3CA gene [24]. In addition, to the best of our knowledge, such extensive ossification has not been seen in previous reports on KTS, so the cause of this series of reactions is worth analyzing.

FoxO1 is a crucial regulator of osteoblast physiology as it is required for proliferation and redox balance in osteoblasts and thereby controls bone formation [25]. Recent research found that FoxO1 provides a favorable intracellular environment for osteoblast functions by defending against the adverse effects of oxidative stress [26]. We utilized NGS analysis to detect a novel homozygous missense variation at FoxO1 in this patient. We hypothesized that due to mutations in FoxO1, the process of favoring protein synthesis and resistance to oxidative stress in osteoblasts was enhanced, promoting bone formation and ossification in the microenvironment of extensive malformed capillaries. In recent studies, it has been confirmed that the expression of FoxO1 is associated with multiple osteogenic phenotypic markers like Runx2 and BMP2, which play an important role in the regulation of osteogenesis [27]. The mechanism of acquired ossification regulated by FoxO1 still requires further studies.

To the best of our knowledge, this is the first reported case of a patient with both KTS and extensive HO, which led to severe lower extremity contracture. This case demonstrates that definitive diagnosis of a combination of these two rare diseases requires detailed imaging analysis and pathologic evidence. Further, it illustrates the challenges of open operation for fractures in KTS patients and the need to anticipate excessive bleeding. Mutations of FoxO1 are the potential regulator of the acquired ossification with KTS, and understanding the exact mechanism requires further research.

## Data Availability

The authors confirm that the data supporting the findings of this study are available within the article.

## Abbreviations

CT: computed tomographic
FoxO: forkhead box O (FoxO) transcription factors
HO: heterotopic ossification
KTS: Klippel-Trenaunay Syndrome
MRI: Magnetic Resonance Imaging
